# Supplementary material for: ZNF185 prevents stress fiber formation through the inhibition of RhoA in endothelial cells
Source: Commun Biol. 2023 Jan 11;6:29. doi: 10.1038/s42003-023-04416-x (PMC9834212; doi:10.1038/s42003-023-04416-x)
Supplement: Supplementary file 2 — Supplementary Information [file 42003_2023_4416_MOESM2_ESM.pdf]

a

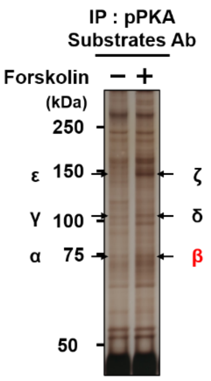

b

Identified Proteins	Accession Number	Molecular Weight	SpC α	SpC β
Zinc finger protein 185<br>OS=Homo sapiens OX=9606 GN=ZNF185 PE=1 SV=3	O15231	74 kDa	0	46
Heat shock protein HSP 90-beta<br>OS=Homo sapiens OX=9606 GN=HSP90AB1 PE=1 SV=4	P08238	83 kDa	5	5
Pre-mRNA-splicing factor ATP-dependent RNA helicase DHX15<br>OS=Homo sapiens OX=9606 GN=DHX15 PE=1 SV=2	O43143	91 kDa	0	3
Tubulin alpha-1A chain<br>OS=Homo sapiens OX=9606 GN=TUBA1A PE=1 SV=1	Q71U36	50 kDa	5	3

Identified Proteins	Accession Number	Molecular Weight	SpC γ	SpC δ
Heat shock cognate 71 kDa protein<br>OS=Homo sapiens OX=9606 GN=HSPA8 PE=1 SV=1	P11142	71 kDa	8	28
Zinc finger protein 185<br>OS=Homo sapiens OX=9606 GN=ZNF185 PE=1 SV=3	O15231	74 kDa	0	15
Stress-70 protein, mitochondrial<br>OS=Homo sapiens OX=9606 GN=HSPA9 PE=1 SV=2	P38646	74 kDa	2	15
Heat shock 70 kDa protein 1A<br>OS=Homo sapiens OX=9606 GN=HSPA1A PE=1 SV=1	P0DMV8	70 kDa	0	13

Identified Proteins	Accession Number	Molecular Weight	SpC ε	SpC ζ
Actin, cytoplasmic 1<br>OS=Homo sapiens OX=9606 GN=ACTB PE=1 SV=1	P60709	42 kDa	0	28
Actin, cytoplasmic 2<br>OS=Homo sapiens OX=9606 GN=ACTG1 PE=1 SV=1	P63261	42 kDa	17	27
Elongation factor 1-alpha 1<br>OS=Homo sapiens OX=9606 GN=EEF1A1 PE=1 SV=1	P68104	50 kDa	7	7
Tubulin alpha-1A chain<br>OS=Homo sapiens OX=9606 GN=TUBA1A PE=1 SV=1	Q71U36	50 kDa	7	6

Supplementary Figure 1 Screening for PKA substrates in HUVECs.

a. Representative image of the silver-stained gel is presented in Fig. 1b. Forskolin (10 μM) was added to HUVECs for 1 h. **b.** Identified proteins by LC-MS/MS. An immunoprecipitated PKA substrate indicated by β was identified as ZNF185. Three bands (β, δ, and ζ) and their control bands (α, γ, and ε) were excised from the gels. SpC: spectral count.

a

The amino acid sequence of human ZNF185 (isoform 4)

1	MSISALGGRT	KGKPLPPGEE	ERNNVLKQMK	VRTTLKGDKS	WITKQDESEG	RTIELPSGRS	RATSFSSAGE	VPKPRPPSTR	APTGYIIRGV	FTKPIDSSSQ
101	PQQQFPKANG	TPKSAASLVR	TANAGPPRPS	SSGYKMTTED	YKKLAPYNIR	RSSTSGDTEE	EEEEEVVPFS	SDEQKRRSEA	ASGVLRRTAP	REHSYVLSAA
201	KKSTGPTQET	QAPFIAKRVE	VVEEDGPSEK	SQDPPALARS	TPGSNSADGG	RTKASRAIWI	ECLPSMPSPA	GSQELSSRGE	EIVRLQILTP	RAGLRLVAPD
301	VEGMRSSPGN	KDKEAPCSRE	LQRDLAGEEA	FRAPNTDAAR	SSAQLSDGNV	GSGATGSRPE	GLAAVDIGSE	RGSSSATSVS	AVPADRKSNS	TAAQEDAKAD
401	PKGALADYEG	KDVATRVGEA	WQERPGAPRG	GQGDPAVPAQ	QPADPSTPER	QSSPSGSEQL	VRRESCGSSV	LTDFEGKDVA	TKVGEAWQDR	PGAPRGGQGD
501	PAVPTQQPAD	PSTPEQQNSP	SGSEQFVRRE	SCTSRVRSPS	SCMVTVTVTA	TSEQPHIYIP	APASELDSSS	TTKGILFVKE	YVNASEVSSG	KPVSARYSNV
601	SSIEDSFAME	KKPPCGSTPY	SERTTGGICT	YCNREIRDCP	KITLEHLGIC	CHEYCFKCGI	CSKPMGDLLD	QIFIHRDTIH	CGKCYEKLF	

b

The amino acid sequence of human ZNF185-ΔPKA

1	MSISALGGRT	KGKPLPPGEE	ERNNVLKQMK	VRTTLKGDKS	WITKQDESEG	RTIELPSGRS	RATSFSSAGE	VPKPRPPSTR	APTGYIIRGV	FTKPIDSSSQ
101	PQQQFPKANG	TPKSAASLVR	TANAGPPRPS	SSGYKMTTED	YKKLAPYNIR	RSSTSGDTEE	EEEEEVVPFS	SDEQKRRSEA	ASGVLRRTAP	REHSYVLSAA
201	KKSTGPTQET	QAPFIAKRVE	VVEEDGPSEK	SQDPPALARS	TPGSNSADGG	RTKASRAIWI	ECLPSMPSPA	GSQELSSRGE	EIVRLQILTP	RAGLRLVAPD
301	VEGMRSSPGN	KDKEAPCSRE	LQRDLAGEEA	FRAPNTDAAR	SSAQLSDGNV	GSGATGSRPE	GLAAVDIGSE	RGSSSATSVS	AVPADRKSNS	TAAQEDAKAD
401	PKGALADYEG	KDVATRVGEA	WQERPGAPRG	GQGDPAVPAQ	QPADPSTPER	QSSPSGSEQL	VRRESCGSSV	LTDFEGKDVA	TKVGEAWQDR	PGAPRGGQGD
501	PAVPTQQPAD	PSTPEQQNSP	SGSEQFVRRE	SCT						
601	SFAME	KKPPCGSTPY	SERTTGGICT	YCNREIRDCP	KITLEHLGIC	CHEYCFKCGI	CSKPMGDLLD	QIFIHRDTIH	CGKCYEKLF	

Supplementary Figure 2 The amino acid sequence of ZNF185.

a, b. ZNF185 isoform 4 and ZNF185-ΔPKA are shown. Three RRXS motifs are indicated in red.

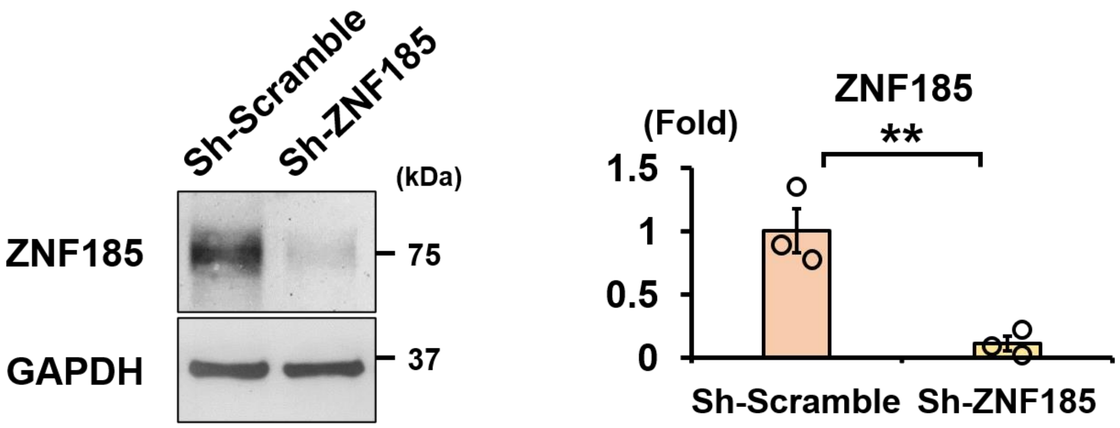

Supplementary Figure 3 The protein expression of ZNF185 is reduced by ZNF185 knockdown.

The endogenous expression of ZNF185 is reduced by ZNF185 knockdown. Representative blots are shown ($n = 3$). Densitometric analysis of ZNF185. $**p < 0.01$ ($n = 3$). Data are presented as the mean ± standard error.

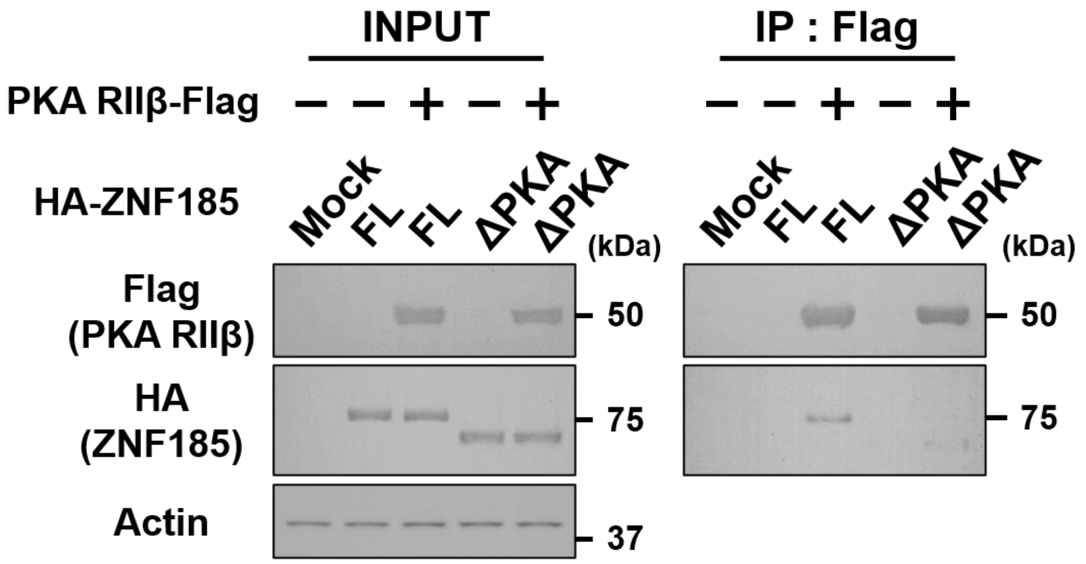

Supplementary Figure 4 PKA RIIβ does not interact with deletion mutant of ZNF185 (Δaa 534-605).

PKA RIIβ-Flag and HA-ZNF185 are overexpressed in HEK293T cells. Anti-Flag beads were used for coimmunoprecipitation. Representative blots are shown (*n* = 3).

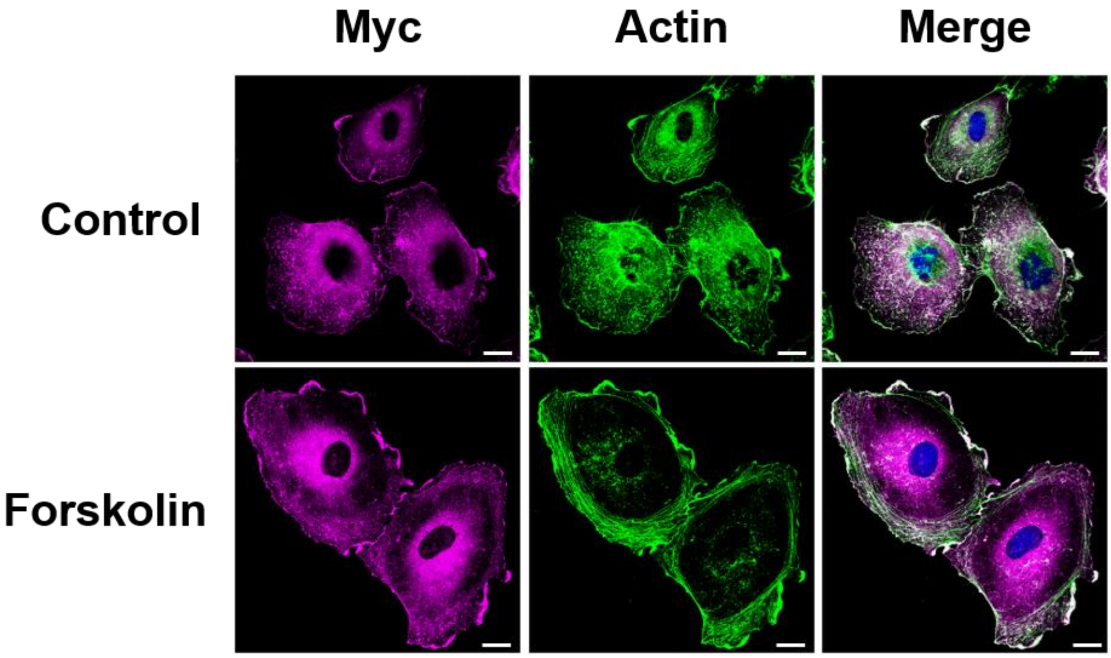

Supplementary Figure 5 ZNF185 colocalizes with actin.

ZNF185 and actin are colocalized. Immunofluorescence staining of Myc (magenta) and actin (green) in HUVECs overexpressing Myc-ZNF185-HA. Forskolin (10 μM) was administered to HUVECs for 1h. Representative images are shown (*n* = 3). Scale bars, 10 μm.

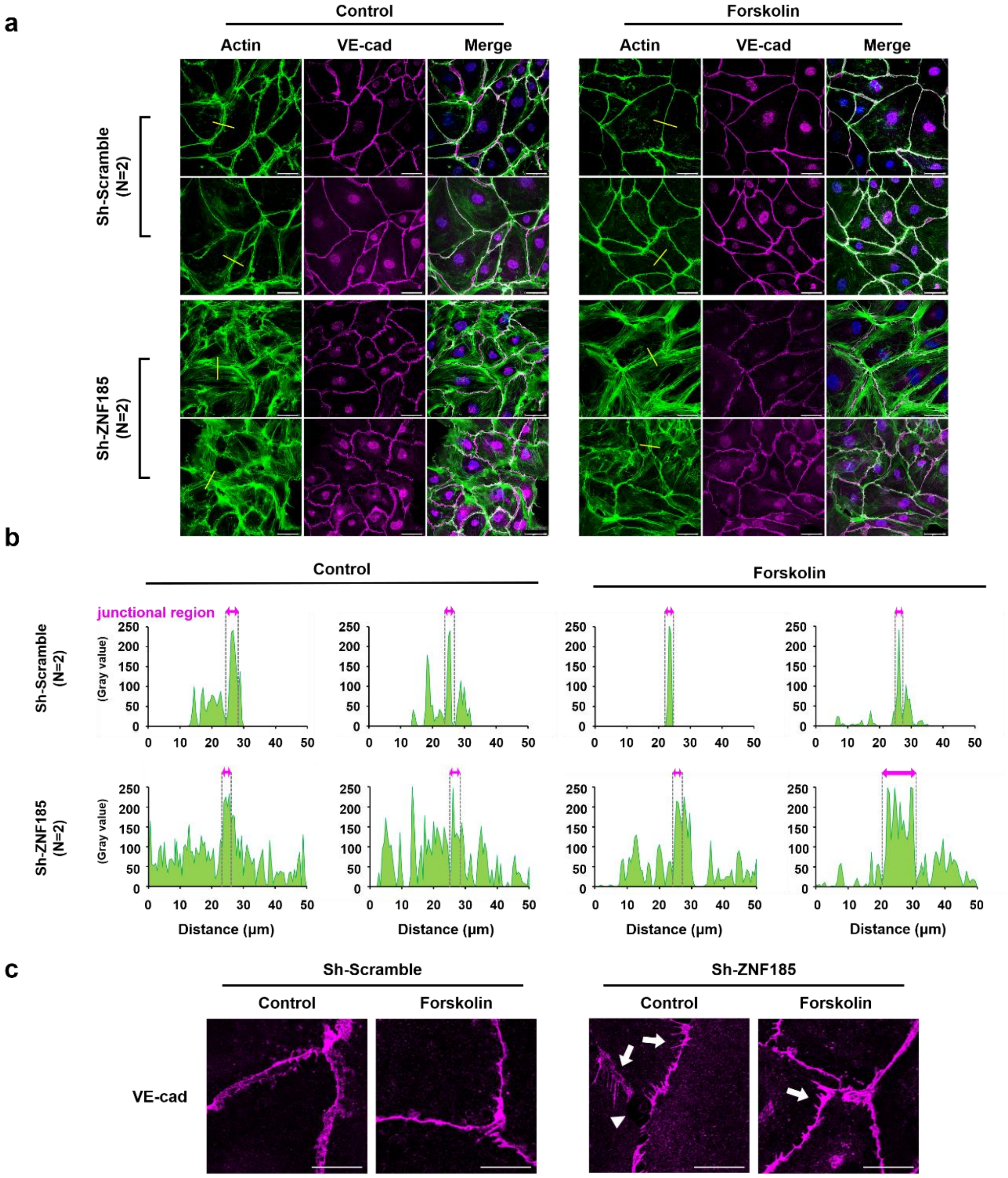

Supplementary Figure 6 ZNF185 knockdown-induced stress fiber formation and discontinuous junctions.

a, b. ZNF185 knockdown induces stress fiber formation and discontinuous cell-cell junctions. **a**. Multiple views from two independent experiments are shown.

Immunofluorescence staining of actin (green) and VE-cadherin (magenta). Scale bars, 50 μm. **b.** Intensity of stress fibers at the yellow lines was quantified. **c.** Enlarged views of fig. 3b are shown. Immunofluorescence staining of VE-cadherin (magenta). Arrows indicate discontinuous zig-zag junctions. Arrowheads indicate intercellular gaps. Scale bars, 25 μm.

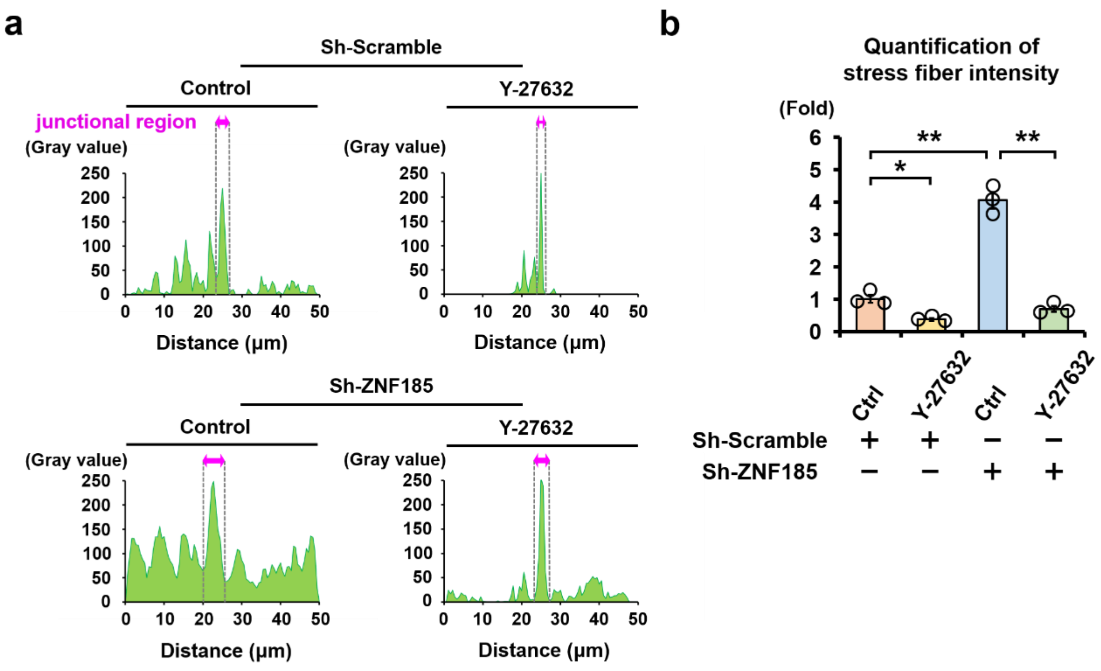

Supplementary Figure 7 Quantification of stress fibers in Fig. 4d.

a. Intensity of stress fiber formation at the yellow lines in Fig. 4d was quantified. **b.** The bar graph shows total amount of stress fiber intensity in Supplementary Fig. 7a. Two-sided Student's t test, $*p < 0.05$, $**p < 0.01$ ($n = 3$). Data are presented as the mean ± standard error. Ctrl: control.

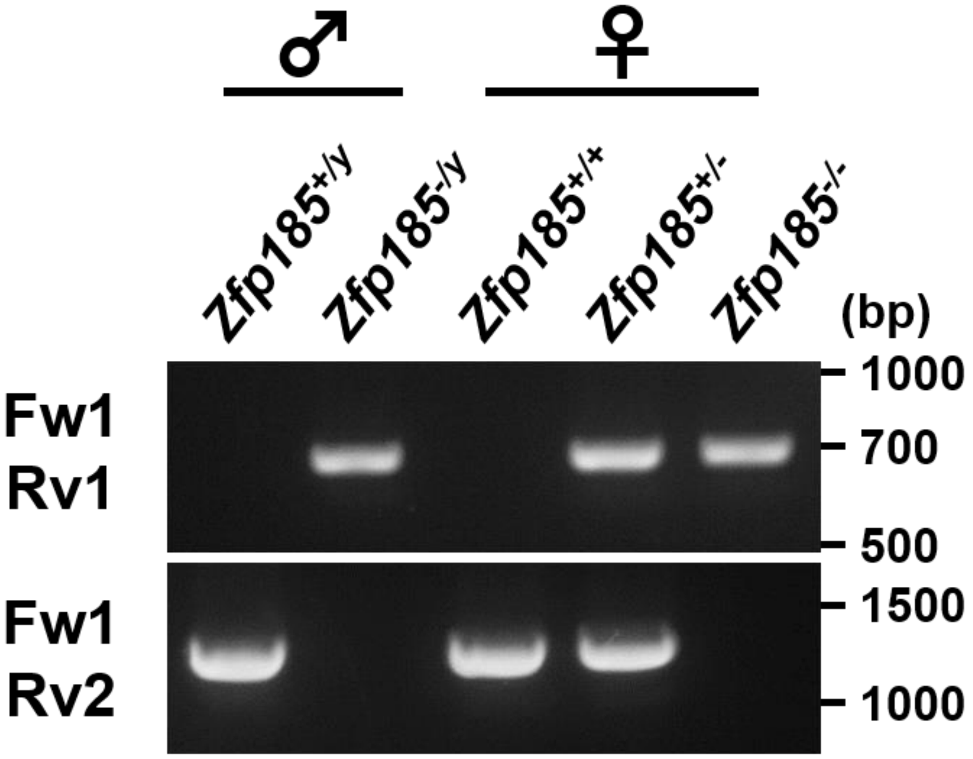

Supplementary Figure 8 Genotyping of *Zfp185* knockout mice.

Zfp185 gene is amplified by PCR from genomic DNA of *Zfp185*$^{+/y}$, *Zfp185*$^{-/y}$, *Zfp185*$^{+/+}$, *Zfp185*$^{+/-}$, *Zfp185*$^{-/-}$ mice.

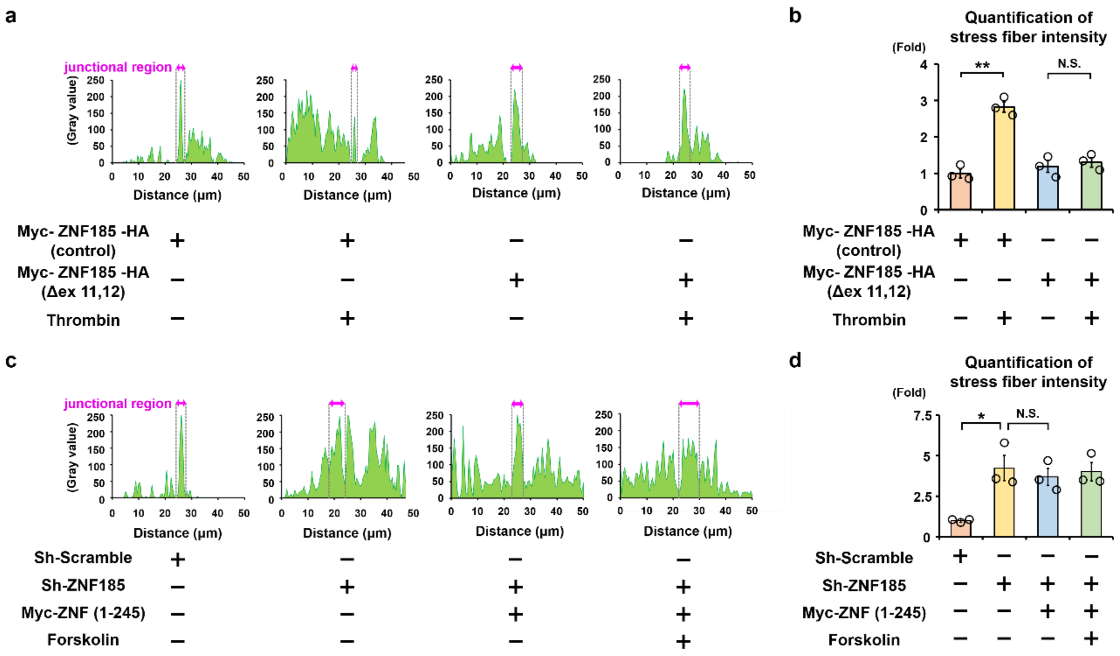

Supplementary Figure 9 Quantification of stress fibers in Fig. 8.

a, b. Quantification of stress fibers in Fig. 8a. **a.** Stress fiber formation at the yellow lines was quantified. **b**. The bar graph shows total amount of stress fiber intensity in Supplementary Fig. 9a. Two-sided Student's t test, $^{**}p < 0.01$ ($n = 3$). **c, d.** Quantification of stress fibers in Fig. 8e. **c.** Stress fiber formation at the yellow lines was quantified. **d.** The bar graph shows total amount of stress fiber intensity in Supplementary Fig. 9c. Two-sided Student's t test, $^{*}p < 0.05$ ($n = 3$). Data are presented as the mean ± standard error.

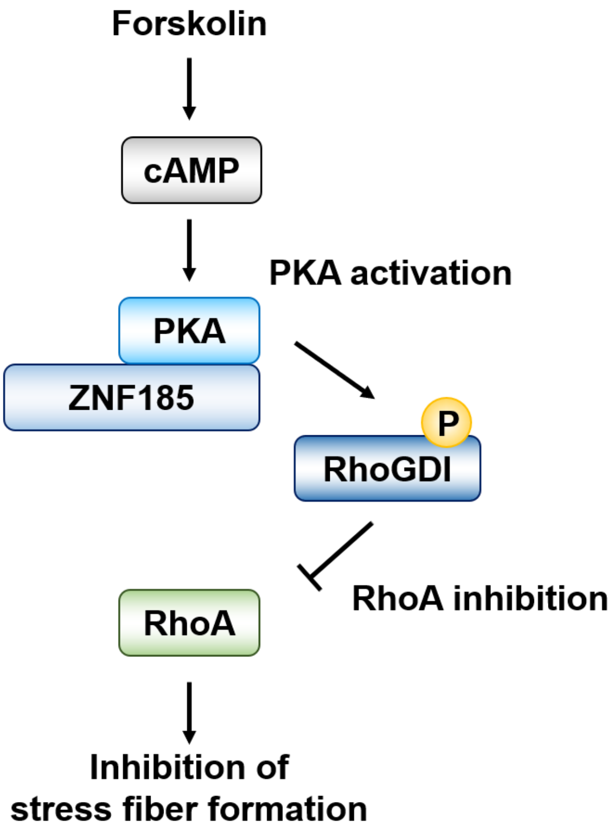

Supplementary Figure 10 A schematic signaling pathway of cAMP/PKA/RhoA.

Forskolin increases intracellular cAMP levels and activates PKA. ZNF185 mediates PKA-induced phosphorylation of RhoGDIα at S174 leading to inhibition of RhoA activity and stress fiber formation.

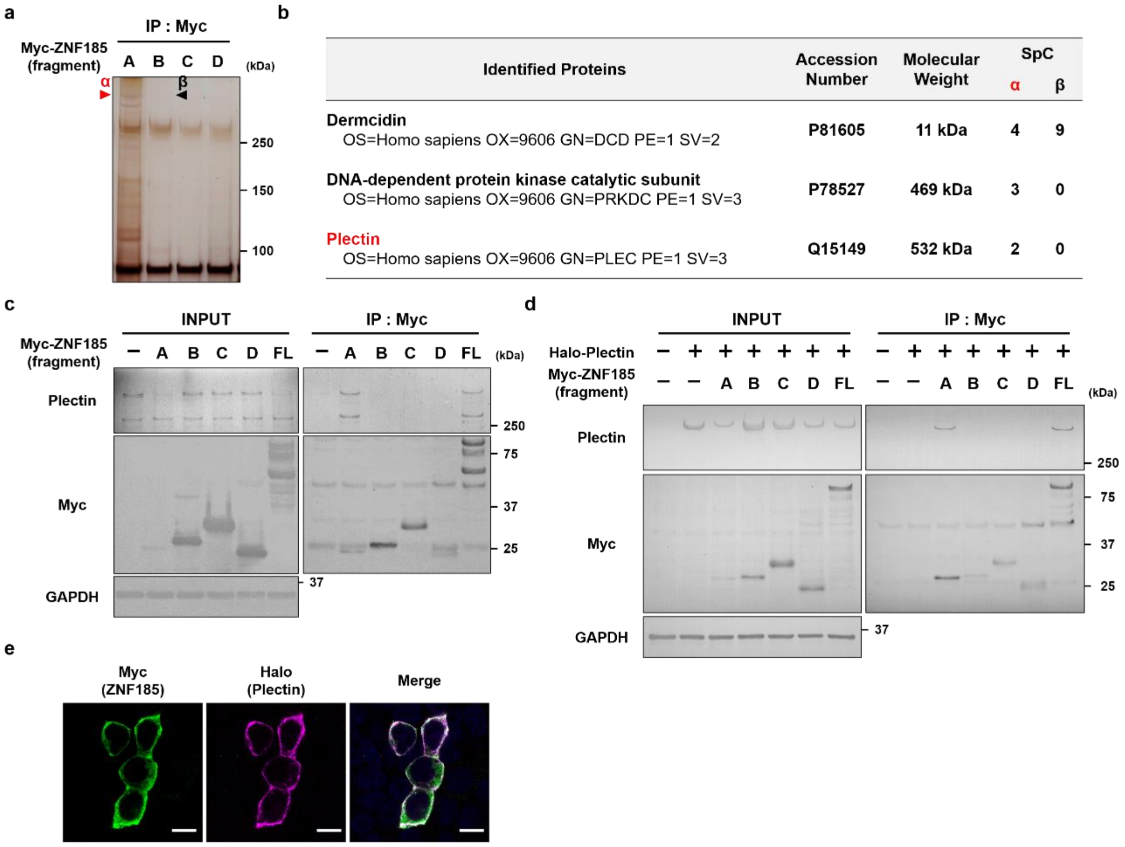

Supplementary Figure 11 Identification of plectin as a ZNF185 interacting protein.

a. Representative silver staining of ZNF185 interacting proteins. The Myc-ZNF185 fragments indicated in Fig. 2c are overexpressed in HEK293T cells. Anti-Myc beads were used for coimmunoprecipitation. ZNF185 fragment A interacts with a protein indicated by α. **b** Identified proteins by LC-MS/MS. SpC: spectral count. **c**, **d.** Plectin interacts with the N-terminal region of ZNF185. **c.** Endogenous plectin interacts with ZNF185 fragment A and full-length ZNF185. Anti-Myc beads were used for coimmunoprecipitation. Representative blots are shown ($n = 3$). **d**. Overexpressed plectin interacts with ZNF185 fragment A and full-length ZNF185. Halo-plectin, Myc-ZNF185 fragments, and full-

length ZNF185 were overexpressed in HKE293T cells. Anti-Myc beads were used for coimmunoprecipitation. Representative blots are shown (n = 3). e. ZNF185 and plectin are colocalized. Immunofluorescence image of Myc (green) and Halo (magenta) in HEK293T overexpressing Myc-ZNF185 and Halo-plectin. Representative images are shown (n = 3). Scale bars, 10 μm.

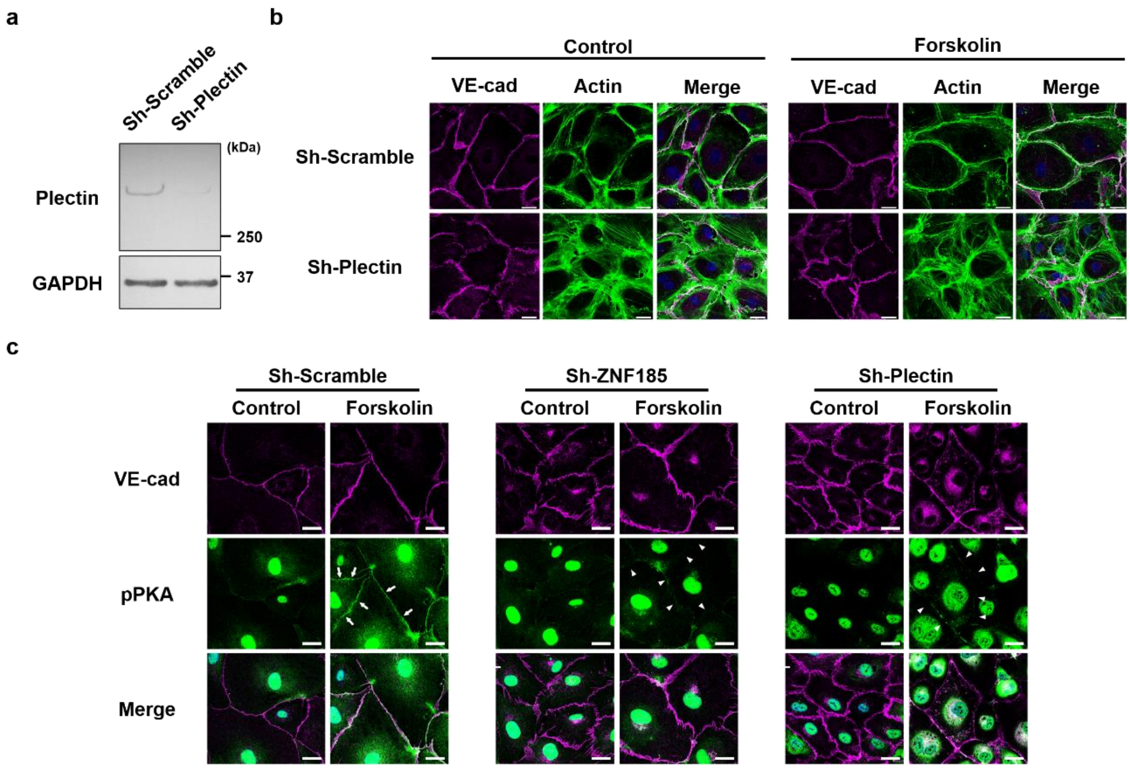

Supplementary Figure 12 Knockdown of plectin induces stress fiber formation and impairs phosphorylation of PKA substrates.

a. The protein expression of plectin is reduced by sh-plectin. Western blot analysis of plectin. Representative blots are shown (n = 3). **b.** Plectin knockdown induces stress fiber formation and discontinuous cell-cell junctions in a confluent HUVEC monolayer. Immunofluorescence staining of VE-cadherin (magenta) and actin (green). Representative images are shown (n = 3). Scale bars, 25 μm. **c.** Knockdown of ZNF185 or plectin inhibits phosphorylation of PKA substrates at the membrane region. Immunofluorescence staining of pPKA in the presence or absence of 10 μM forskolin in HUVECs transduced with scrambled, ZNF185, or plectin shRNA. Arrows indicate

phosphorylated PKA substrates at the membrane region. Arrowheads indicate the impairment of phosphorylation of PKA substrates. Immunofluorescence staining of VE-cadherin (magenta) and pPKA (green). Representative images are shown (*n* = 3). Scale bars, 25 μm.

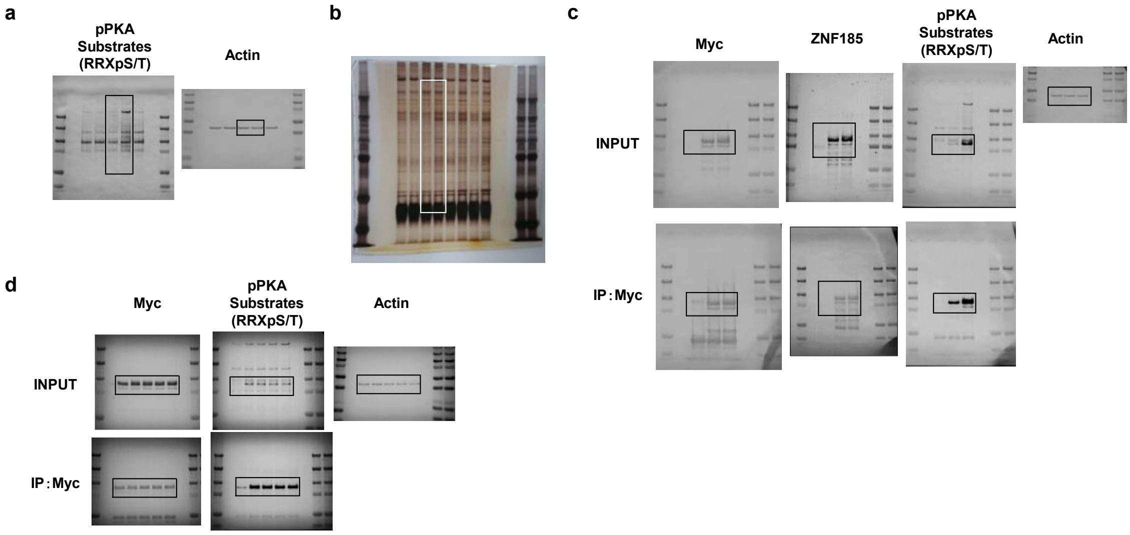

Supplementary Figure 13 Uncropped western blots of Figure 1.

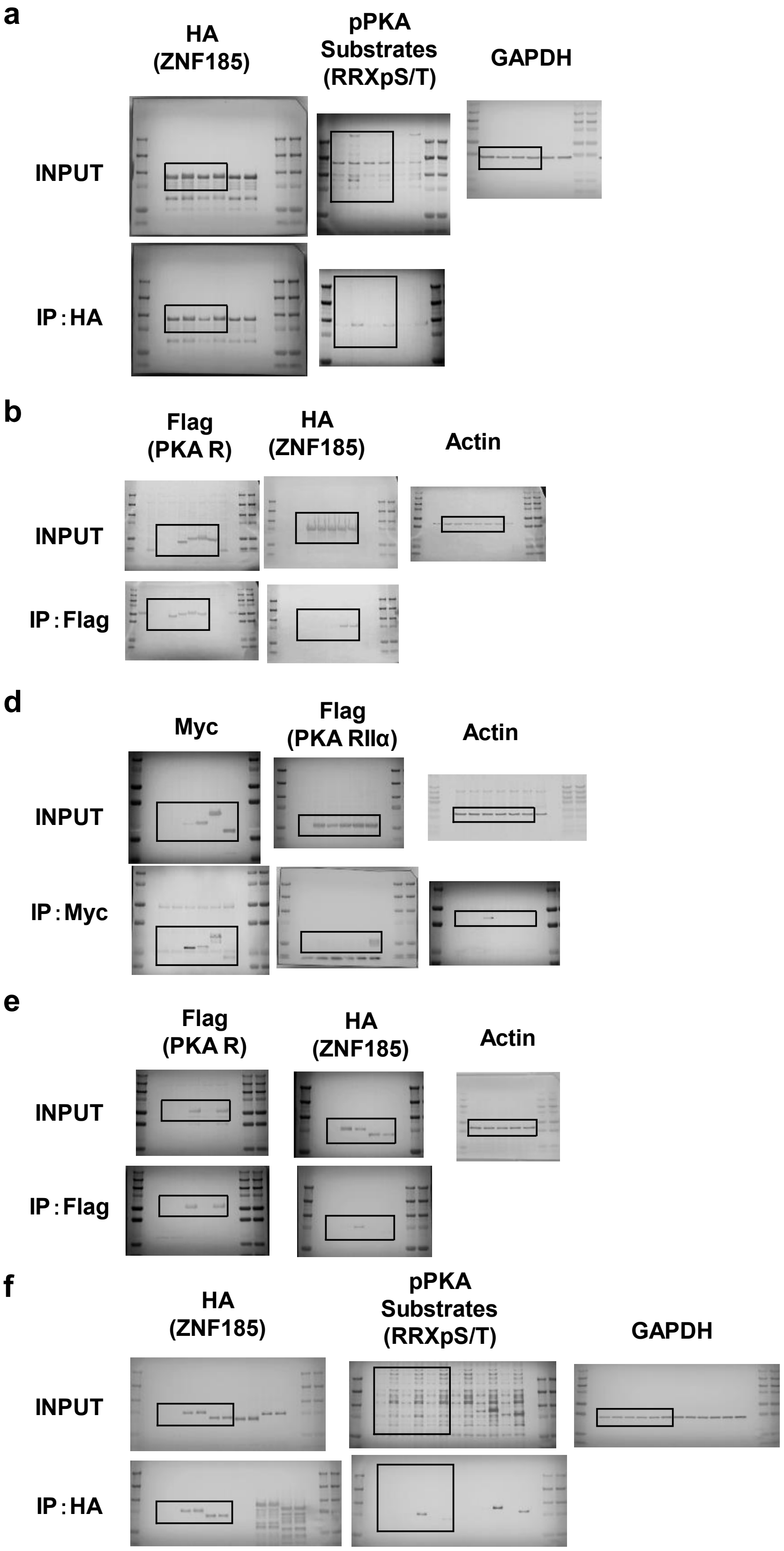

Supplementary Figure 14 Uncropped western blots of Figure 2.

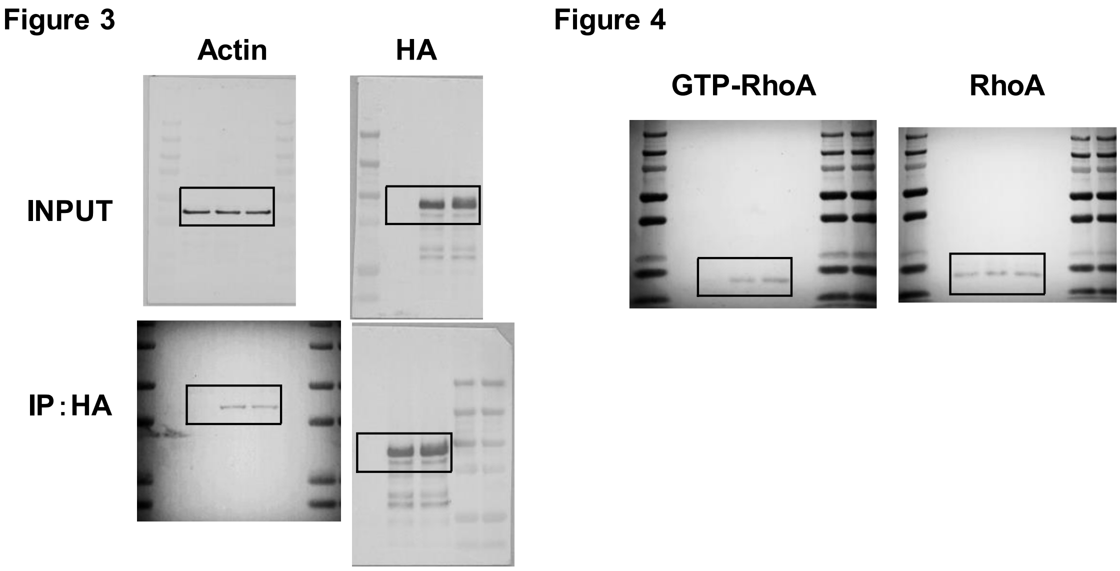

Supplementary Figure 15 Uncropped western blots of Figure 3 and 4.

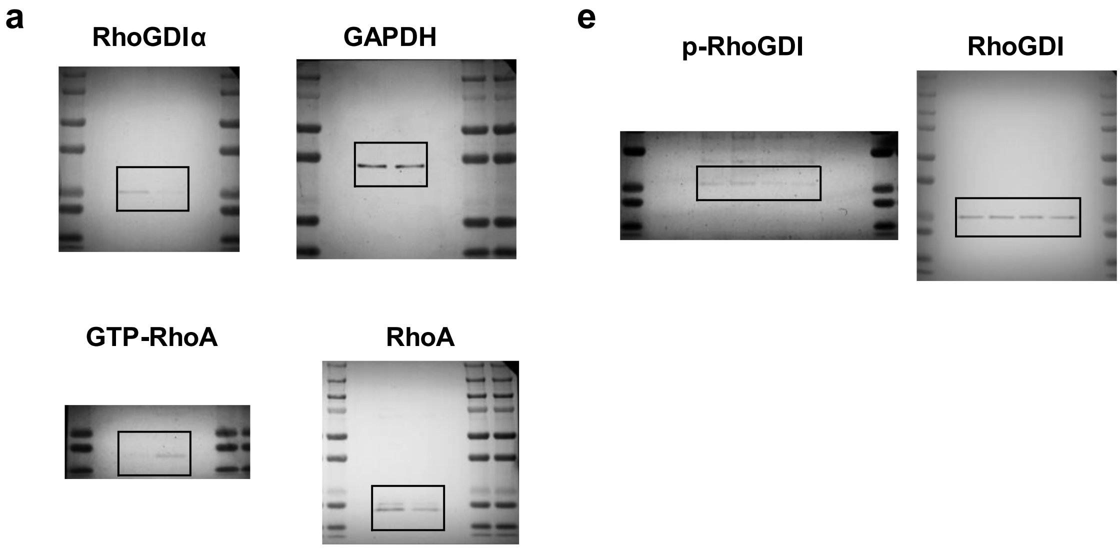

Supplementary Figure 16 Uncropped western blots of Figure 5.

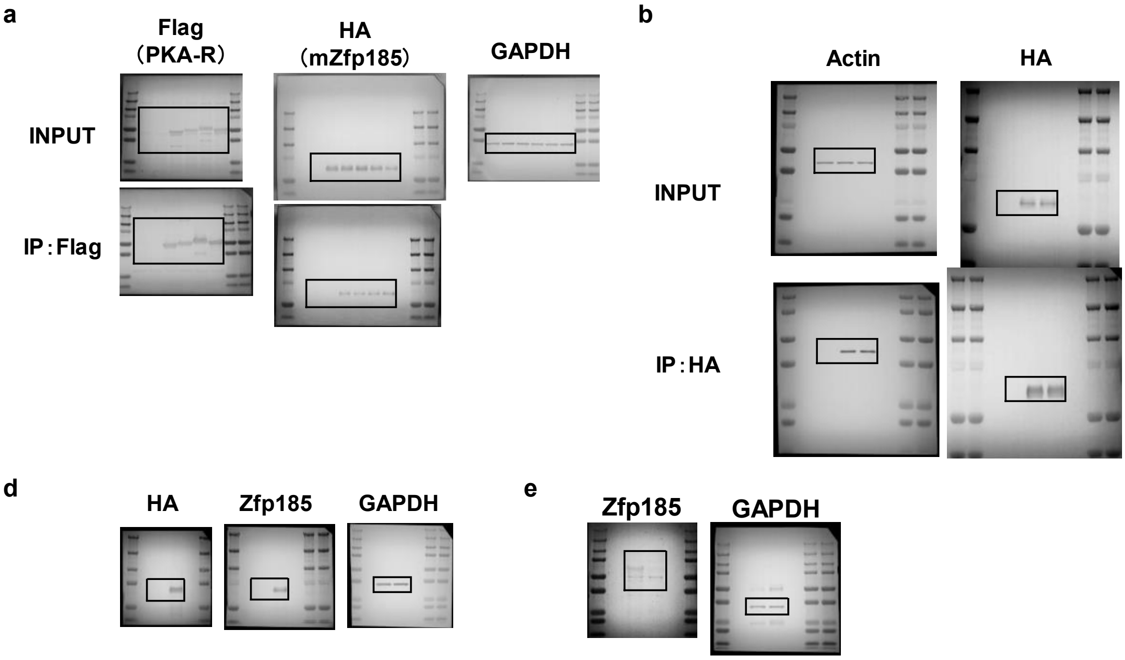

Supplementary Figure 17 Uncropped western blots of Figure 6.

Figure 7

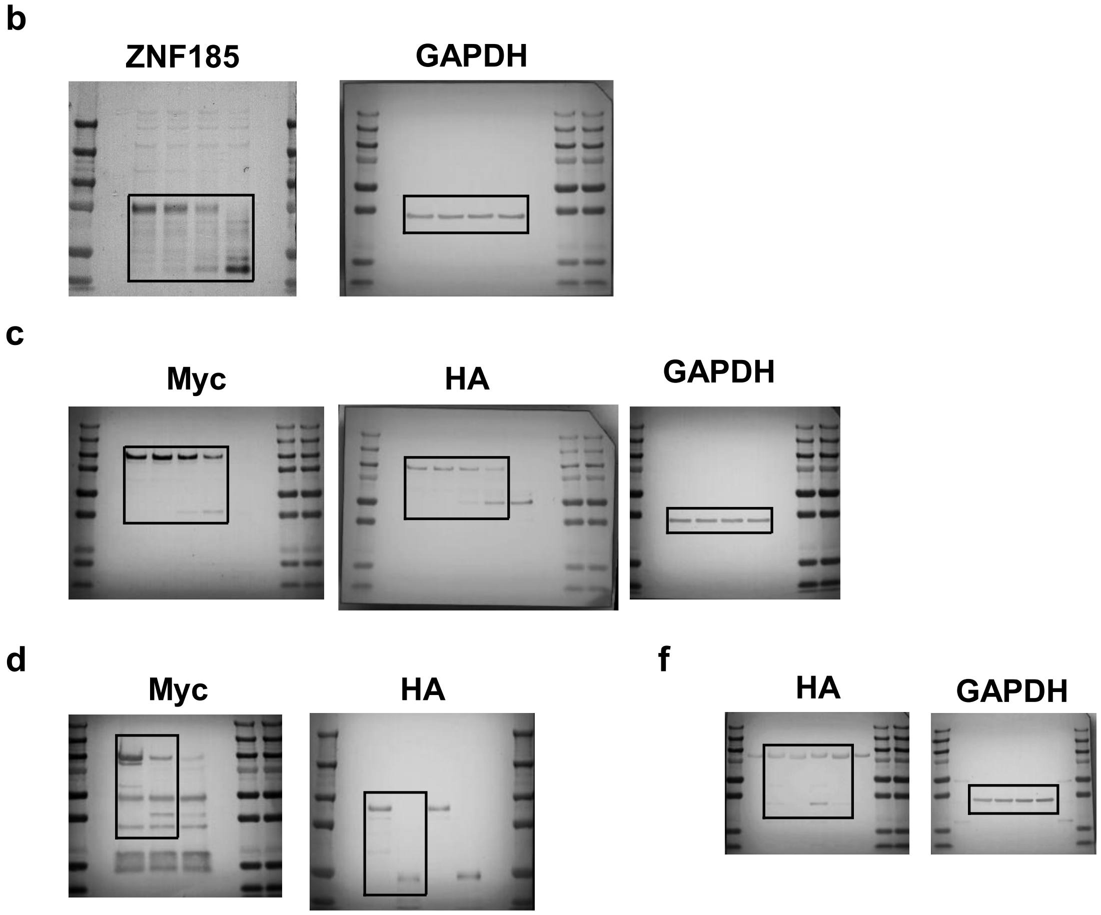

Figure 8

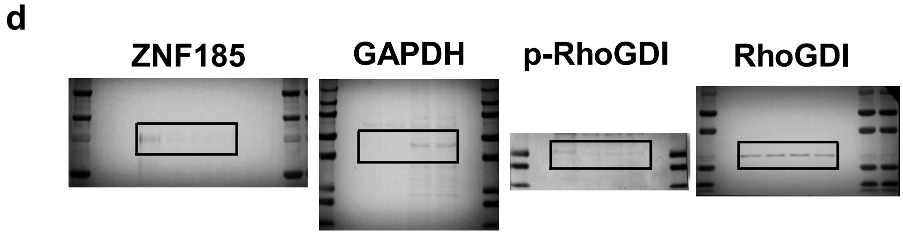

Supplementary Figure 18 Uncropped western blots of Figure 7 and 8.

Supplementary Figure 3

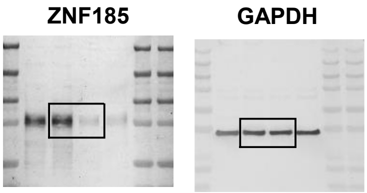

Supplementary Figure 4

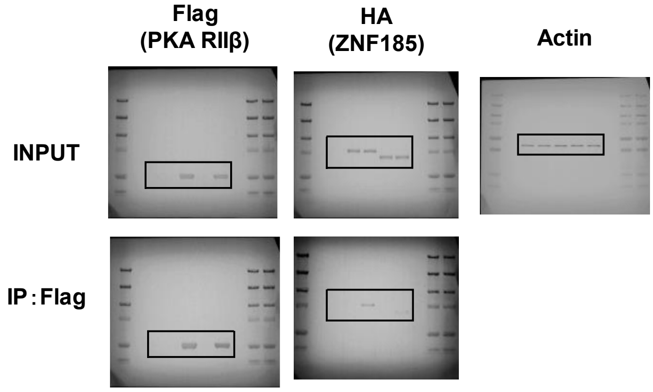

Supplementary Figure 8

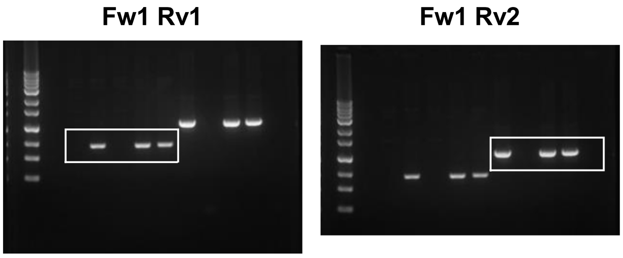

Supplementary Figure 19 Uncropped western blots of Supplementary Figure 3, 4, and 8.

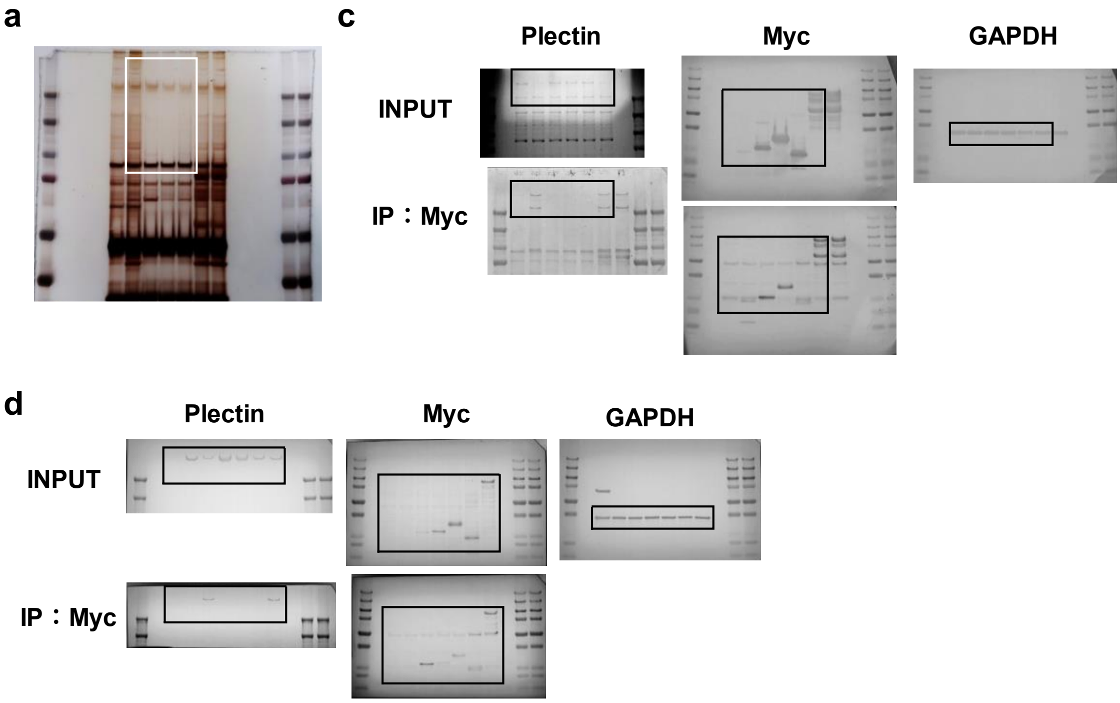

Supplementary Figure 20 Uncropped western blots of Supplementary Figure 11.

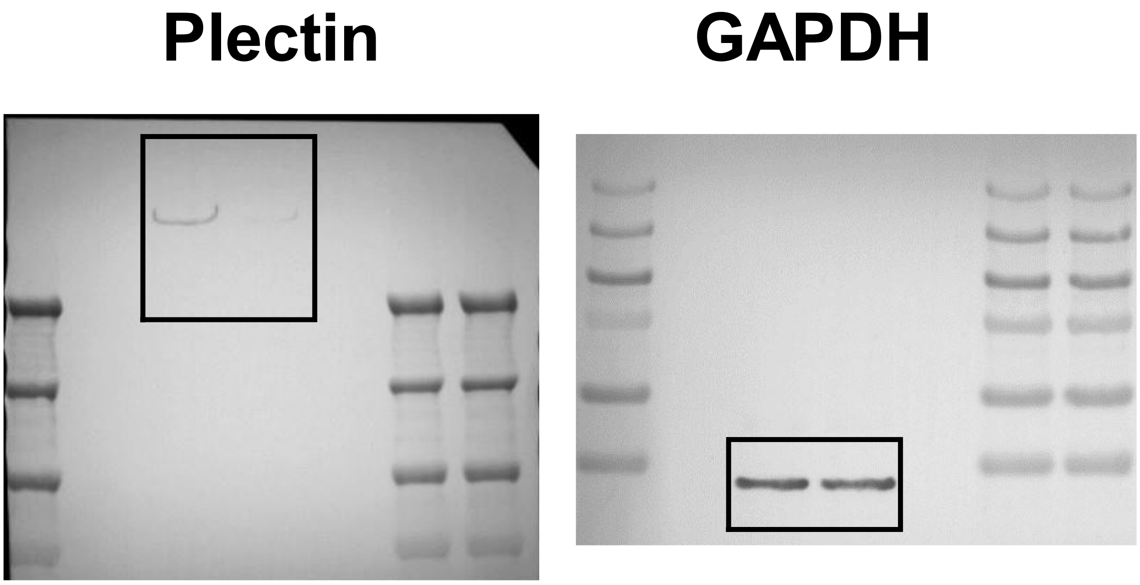

Supplementary Figure 21 Uncropped western blots of Supplementary Figure 12.

Gene	Forward primer (5'→3')	Reverse primer (3'→5')
**Myc-ZNF185-HA**	AATTCAGTCGACTGGATCCGGTACCATGGGAGAACAAAAGTTGATTTCTGAAGAAGATTTGGGAAGCGAACAAAAGTTGATTTCTGAAGAAGATTTGGGAAGCGAACAAAAGTTGATTTCTGAAGAAGATTTGGGAAGCAGTATCTCAGCTCTT	GTACAAGAAAGCTGGGTCTAGATATCTCTAAGCGTAATCCGGAACATCATACGGGTAGCTTCCAGCGTAATCCGGAACATCATACGGGTAGCTTCCAGCGTAATCCGGAACATCATACGGGTAGAAGAGCTTCTCATAGCA
**Myc-ZNF185-Δexon 11,12-HA**	TCCACTCCTGGCTCAAACAGGTCTTCCCCAGGCAAC	GTTGCCTGGGGAAGACCTGTTTGAGCCAGGAGTGGA
**Myc-N-terminal ZNF185**	AATTCAGTCGACTGGATCCGGTACCATGGGAGAACAAAAGTTGATTTCTGAAGAAGATTTGGGAAGCGAACAAAAGTTGATTTCTGAAGAAGATTTGGGAAGCGAACAAAAGTTGATTTCTGAAGAAGATTTGGGAAGCAGTATCTCAGCTCTT	GTACAAGAAAGCTGGGTCTAGATATCTCTAGTTTGAGCCAGGAGTGGA
**C-terminal ZNF185-HA**	TCGACTGGATCCGGTACCATGAGGTCTTCCCCAGGCAAC	GTACAAGAAAGCTGGGTCTAGATATCTCTAAGCGTAATCCGGAACATCATACGGGTAGCTTCCAGCGTAATCCGGAACATCATACGGGTAGCTTCCAGCGTAATCCGGAACATCATACGGGTAGAAGAGCTTCTCATAGCA

Supplementary Table 1 Primer sequences.
