# Supplementary material for: ZNF185 prevents stress fiber formation through the inhibition of RhoA in endothelial cells
Source: Commun Biol. 2023 Jan 11;6:29. doi: 10.1038/s42003-023-04416-x (PMC9834212; doi:10.1038/s42003-023-04416-x)
Supplement: Supplementary file 3 — Description of Additional Supplementary Files [file 42003_2023_4416_MOESM3_ESM.pdf]

Description of Additional Supplementary Files

File name: Supplementary Data 1
Description: The numerical source data for graphs and charts.
