# Supplementary material for: ZNF185 prevents stress fiber formation through the inhibition of RhoA in endothelial cells
Source: Commun Biol. 2023 Jan 11;6:29. doi: 10.1038/s42003-023-04416-x (PMC9834212; doi:10.1038/s42003-023-04416-x)
Supplement: Supplementary file 5 — Reporting Summary [file 42003_2023_4416_MOESM5_ESM.pdf]

nature portfolio

Corresponding author(s): Fumiaki Ando

Last updated by author(s): Dec 5, 2022

Reporting Summary

Nature Portfolio wishes to improve the reproducibility of the work that we publish. This form provides structure for consistency and transparency in reporting. For further information on Nature Portfolio policies, see our Editorial Policies and the Editorial Policy Checklist.

Statistics

For all statistical analyses, confirm that the following items are present in the figure legend, table legend, main text, or Methods section.

n/a	Confirmed	
☐	☒	The exact sample size (*n*) for each experimental group/condition, given as a discrete number and unit of measurement
☐	☒	A statement on whether measurements were taken from distinct samples or whether the same sample was measured repeatedly
☐	☒	The statistical test(s) used AND whether they are one- or two-sided<br>*Only common tests should be described solely by name; describe more complex techniques in the Methods section.*
☒	☐	A description of all covariates tested
☒	☐	A description of any assumptions or corrections, such as tests of normality and adjustment for multiple comparisons
☐	☒	A full description of the statistical parameters including central tendency (e.g. means) or other basic estimates (e.g. regression coefficient) AND variation (e.g. standard deviation) or associated estimates of uncertainty (e.g. confidence intervals)
☐	☒	For null hypothesis testing, the test statistic (e.g. *F*, *t*, *r*) with confidence intervals, effect sizes, degrees of freedom and *P* value noted<br>*Give P values as exact values whenever suitable.*
☒	☐	For Bayesian analysis, information on the choice of priors and Markov chain Monte Carlo settings
☒	☐	For hierarchical and complex designs, identification of the appropriate level for tests and full reporting of outcomes
☒	☐	Estimates of effect sizes (e.g. Cohen's *d*, Pearson's *r*), indicating how they were calculated

Our web collection on statistics for biologists contains articles on many of the points above.

Software and code

Policy information about availability of computer code

Data collection	FilterMax F5 (Molecular Divices), TCS SP8 (Leica)
Data analysis	ImageJ (NIH)

For manuscripts utilizing custom algorithms or software that are central to the research but not yet described in published literature, software must be made available to editors and reviewers. We strongly encourage code deposition in a community repository (e.g. GitHub). See the Nature Portfolio guidelines for submitting code & software for further information.

Data

Policy information about availability of data

All manuscripts must include a data availability statement. This statement should provide the following information, where applicable:

- Accession codes, unique identifiers, or web links for publicly available datasets
- A description of any restrictions on data availability
- For clinical datasets or third party data, please ensure that the statement adheres to our policy

All data are included in the main article and associated files.

Human research participants

Policy information about studies involving human research participants and Sex and Gender in Research.

Reporting on sex and gender	n/a
Population characteristics	n/a
Recruitment	n/a
Ethics oversight	n/a

Note that full information on the approval of the study protocol must also be provided in the manuscript.

Field-specific reporting

Please select the one below that is the best fit for your research. If you are not sure, read the appropriate sections before making your selection.

☒ Life sciences ☐ Behavioural & social sciences ☐ Ecological, evolutionary & environmental sciences

For a reference copy of the document with all sections, see nature.com/documents/nr-reporting-summary-flat.pdf

Life sciences study design

All studies must disclose on these points even when the disclosure is negative.

Sample size	The sample size is not statistically predetermined. We used a generally accepted sample size based on out past experience. For experiments, a minimum of 3 samples were chosen based on the standard practice of the field.
Data exclusions	No data was excluded.
Replication	All data represents at least three independent experiments.
Randomization	Samples were randomly allocated to experimental groups.
Blinding	Experiments were blinded where appropriate.

Reporting for specific materials, systems and methods

We require information from authors about some types of materials, experimental systems and methods used in many studies. Here, indicate whether each material, system or method listed is relevant to your study. If you are not sure if a list item applies to your research, read the appropriate section before selecting a response.

Materials & experimental systems

n/a	Involved in the study	
☐	☒	Antibodies
☐	☒	Eukaryotic cell lines
☒	☐	Palaeontology and archaeology
☐	☒	Animals and other organisms
☒	☐	Clinical data
☒	☐	Dual use research of concern

Methods

n/a	Involved in the study	
☒	☐	ChIP-seq
☒	☐	Flow cytometry
☒	☐	MRI-based neuroimaging

Antibodies

Antibodies used

Primary antibodies:
Cell Signaling Technology
rabbit anti-phospho-PKA substrate (#9624)
mouse anti-Myc-Tag (#9B11)
rabbit anti-HA-Tag (#C29F4)
rabbit anti-RhoA (#2117)
rabbit anti-β-actin (13E5)(#4970)

rabbit anti-Myc-tag (71D10) (#2278)

Sigma-Aldrich Corporation
mouse anti-β-actin (#A2228)
mouse anti-Flag (M2) (#F3165)
rabbit anti-ZNF185 (#HPA000400)

Santa Cruz
mouse anti-GAPDH (#sc-32233)

Abcam
rabbit anti-RhoGDI (#ab133248)

Assay Biotechnology Company
rabbit anti-ARHGDIA (Phospho-Ser174) (#A1189)

Bio-Rad
rabbit anti-Plectin (#VPA00847)

Secondary antibodies:
Promega Corporation
anti-Rabbit IgG (Fc), AP Conjugate (#S3731)
anti-Mouse IgG (H+L), AP Conjugate (#S3721)

Sigma-Aldrich Corporation
goat anti-mouse light-chain antibody (#AP200A)

Validation

Antibodies used were all reported and validated by the manufacturer and in the literature.
rabbit anti-phospho-PKA substrate (#9624; Cell Signaling Technology) https://www.cellsignal.com/products/primary-antibodies/phospho-pka-substrate-rrxs-t-100g7e-rabbit-mab/9624
mouse anti-Myc-Tag (#9B11; Cell Signaling Technology) https://www.cellsignal.jp/products/primary-antibodies/myc-tag-9b11-mouse-mab/2276
rabbit anti-HA-Tag (#C29F4; Cell Signaling Technology) https://www.cellsignal.jp/products/primary-antibodies/ha-tag-c29f4-rabbit-mab/3724
rabbit anti-RhoA (#2117; Cell Signaling Technology) https://www.cellsignal.com/products/primary-antibodies/rhoa-67b9-rabbit-mab/2117
rabbit anti-β-actin (#4970; Cell Signaling Technology) https://www.cellsignal.jp/products/primary-antibodies/b-actin-13e5-rabbit-mab/4970
rabbit anti-Myc-tag (71D10) (#2278; Cell Signaling Technology) https://www.cellsignal.jp/products/primary-antibodies/myc-tag-71d10-rabbit-mab/2278?_=1666777409603&Ntt=71d10&tahead=true
mouse anti-β-actin (#A2228; Sigma–Aldrich Corporation) https://www.sigmaaldrich.com/JP/ja/product/sigma/a2228
mouse anti-Flag (M2) (#F3165, Sigma-Aldrich Corporation) https://www.sigmaaldrich.com/JP/ja/product/sigma/f3165
rabbit anti-ZNF185 (#HPA000400; Sigma-Aldrich Corporation) https://www.sigmaaldrich.com/JP/ja/product/sigma/hpa000400
mouse anti-GAPDH (#sc-32233; Santa Cruz) https://www.scbt.com/p/gapdh-antibody-6c5
rabbit anti-RhoGDI (#ab133248; Abcam) https://www.abcam.co.jp/rhogdi-antibody-epr3773-ab133248.html
rabbit anti-ARHGDIA (Phospho-Ser174) (#A1189; Assay Biotechnology Company) https://www.assaybiotechnology.com/ARHGDIA-Phospho-Ser174-Antibody-A1189-WB-IHC-ELISA
rabbit anti-Plectin (#VPA00847; Bio-Rad Laboratories) https://www.bio-rad-antibodies.com/polyclonal/human-plectin-antibody-vpa00847.html?f=purified
Anti-Rabbit IgG (Fc), AP Conjugate (#S3731; Promega Corporation) https://www.promega.jp/products/protein-detection/primary-and-secondary-antibodies/anti-rabbit-igg-fc-ap-conjugate/?catNum=S3731
Anti-Mouse IgG (H+L), AP Conjugate (#S3721; Promega Corporation) https://www.promega.jp/products/protein-detection/primary-and-secondary-antibodies/anti-mouse-igg-h-and-l-ap-conjugate/?catNum=S3721
goat anti-mouse light-chain antibody (#AP200A; Sigma-Aldrich) https://www.sigmaaldrich.com/JP/ja/product/mm/ap200a

Eukaryotic cell lines

Policy information about cell lines and Sex and Gender in Research

Cell line source(s)

Human umbilical vein endothelial cells (HUVECs) were obtained from PromoCell, and Human embryonic kidney 293T (HEK293T) cells were obtained from American Type Culture Collection (ATCC).

Authentication

All cell lines were commonly used cell lines purchesed from PromoCell and ATCC.

Mycoplasma contamination

All cell lines were negative for mycoplasma contamination.

Commonly misidentified lines
(See ICLAC register)

No commonly misidentified cell lines were used.

Animals and other research organisms

Policy information about studies involving animals; ARRIVE guidelines recommended for reporting animal research, and Sex and Gender in Research

Laboratory animals	We used genetically modified mice (mus musculus) for this study. All mouse lines have been backcrossed to C57BL/6 background. Male mice were used for experiments.
Wild animals	n/a
Reporting on sex	n/a
Field-collected samples	n/a
Ethics oversight	All animal studies were performed in accordance with the guidelines for animal research of Tokyo Medical and Dental University. The study protocol was approved by the Animal Care and Use Committee of Tokyo Medical and Dental University (approval number: A2021-120C).

Note that full information on the approval of the study protocol must also be provided in the manuscript.
